# The incidence and interrelationship of hemivertebra and concomitant cardiac abnormalities in congenital scoliosis

**DOI:** 10.1186/s12891-023-06727-w

**Published:** 2023-07-31

**Authors:** Shengru Wang, Yiwei Zhao, You Du, Jianguo Zhang, Bin Yu

**Affiliations:** Department of Orthopedic Surgery, Peking Union Medical College Hospital, Chinese Academy of Medical Sciences and Peking Union Medical College, 1st Shuai Fu Yuan, Dongcheng District, Beijing, 100730 P. R. China

**Keywords:** Congenital scoliosis, Hemivertebra, Ultrasonic cardiography, Cardiac abnormalities

## Abstract

**Background:**

Congenital scoliosis(CS) is associated with multiple organs defect, and cardiac abnormalities have been reported commonly associated with CS. Hemivertebra is caused by the failure of vertebral formation, which is a major constitute of CS. Till now, few studies focus on the incidence and interrelationship of hemivertebra and concomitant cardiac abnormalities in congenital scoliosis. We aimed to analyze the cardiac defect in CS patients with or without hemivertebra, and further explore the incidence of cardiac defect between different types of hemivertebra.

**Methods:**

The ultrasonic cardiography (UCG) results of surgically treated congenital scoliosis (CS) patients between 2015 and 2018 were retrospectively analyzed. Patients were divided into hemivertebra group and non-hemivertebra group according to preoperative CT. Patients with hemivertebra was further divided into sub-group by single/multiple or fully/partially/mixed segmented hemivertebra. Demographic information, radiographic data and cardiac abnormalities were statistically compared between groups.

**Results:**

A total of 329 patients were analyzed, including 216 patients with hemivertebra and 113 patients without hemivertebra. UCG results were abnormal in 89 cases (27.1%), including 41 males(12.5%) and 48 females(14.6%). Hemivertebra group had comparable incidence of cardiac abnormalities with non-hemivertebra group (p = 0.517). No significant difference in the incidence of UCG abnormalities between single and multiple hemivertebra group (P = 0.246). Binary logistic regression analysis showed that female sex with multiple hemivertebra was a risk factor for abnormal UCG (P = 0.009, OR = 3.449). Cardiac abnormalities was comparable among fully, partially and mixed segmented hemivertebra group(P = 0.264). In abnormal UCG, 33 patients with hemivertebra had non-valvular abnormalities, and 48.5% (16/33) were septal defects. 28 patients had valvular abnormalities, most of them were mitral valve abnormalities, especially mitral valve redundancy, prolapse and insufficiency(82.1%, 23/28). No significant difference between the incidence of non-valvular and valvular abnormalities in patients with hemivertebra (P = 0.581).

**Conclusions:**

The incidence of abnormal UCG results was approximately 28.2% in CS patients with hemivertebra. Female patients with multiple hemivertebra had a higher risk of UCG abnormalities. Mitral valve abnormalities were the most common abnormality of UCG found in CS patients with hemivertebra.

**Trial registration:**

retrospectively registered.

## Background

Hemivertebra are caused by one side of the vertebral body developing while the other side does not during embryonic development, and its incidence is approximately 1–10/per 1000 live infants [1–5]. Hemivertebra are rare spinal deformities that can lead to scoliosis during growth. It can be independent vertebral malformations or a part of genetic syndromes, including Jarcho-Levin syndrome, Klippel-Feil syndrome and VACTERL syndrome [4, 6–10]. Malformation of vertebra is often associated with other skeletal abnormalities, such as ribs and limbs. In addition, due to the common origin of embryonic development, abnormalities of other organs and systems derived from mesoderm development can arise concurrently, among which cardiac and genitourinary tract abnormalities are common extraskeletal abnormalities of hemivertebra [1, 3–5]. Basu et al. [1] used ultrasonic cardiography to investigate patients with congenital scoliosis and found that 26% of the patients had cardiac abnormalities. Goldstein et al. [1]. found cardiac abnormalities in 8% of hemivertebra patients. However, the detail of ultrasonic cardiographic results in different types of hemivertebra is lack. Single or multiple, fully or partially segmented hemivertebra may indicate different dysplasia in embryonic development, which may also associated with extraskeletal abnormalities. The current study intends to analyze the cardiac defect in CS patients with or without hemivertebra, and further explore the incidence of cardiac defect between different types of hemivertebra.

## Methods

### Participants and demographics

Institutional Review Board (IRB) approval of our institution was obtained for the study. We retrospectively reviewed the medical records and radiographic data of CS patients who underwent posterior spinal correction surgery in our hospital between January 2015 and December 2018. The inclusion criteria were as follows: (1) age ≤ 18 years; (2) full clinical and radiographic data from which we could determine the classification of the patients’ hemivertebra; and (3) detailed ultrasonic cardiography (UCG) examination results.

**Fig. 1:**
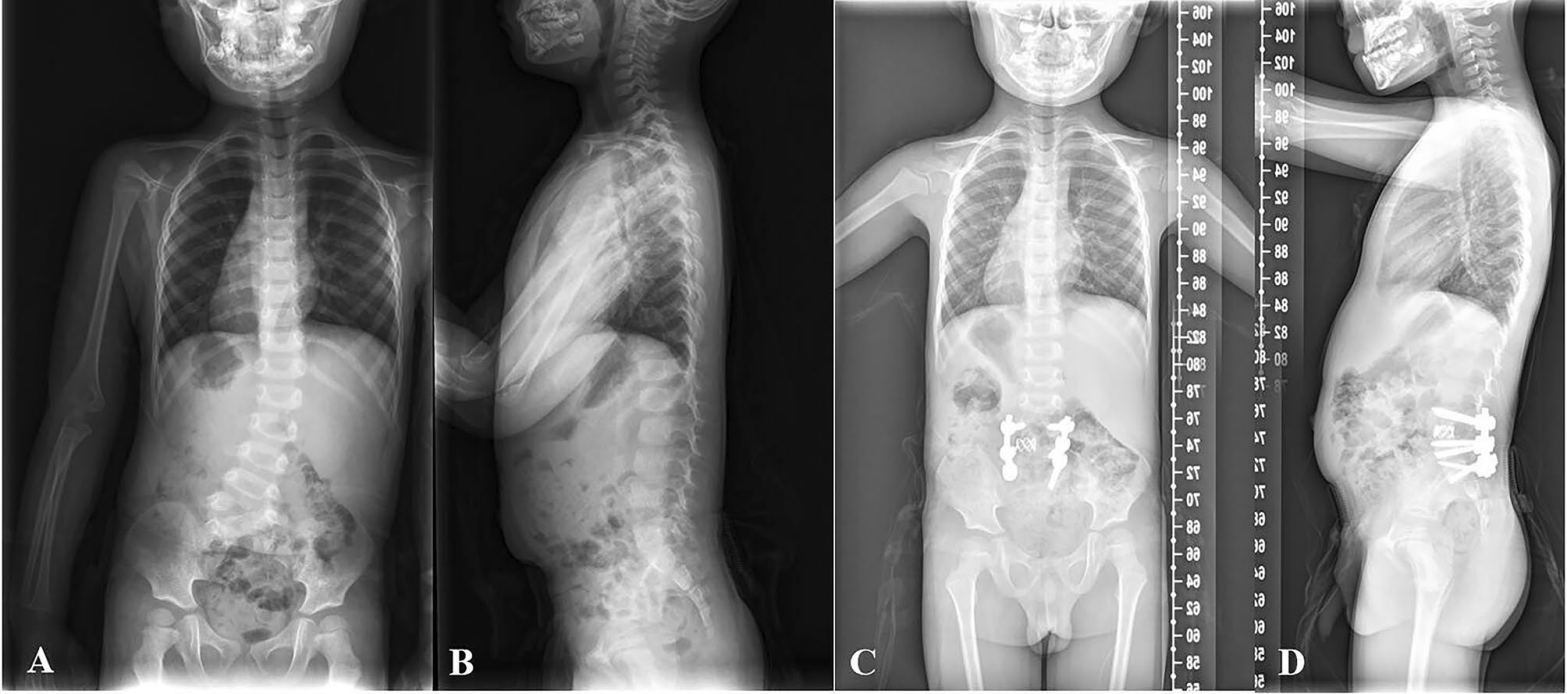
A case of fully segmented hemivertebra with concomitant cardiac abnormalities A 4/M, diagnosed with congenital scoliosis, he had a fully segmented hemivertebra at L4/5 segment **(a, b).** The ultrasonic cardiography showed that he also had mitral valve insufficiency. And he underwent posterior hemivertebra resection and short segment fusion surgery, the X-ray showed L4/5 hemivertebra was totally resected and well deformity correction **(c, d)**

**Fig. 2:**
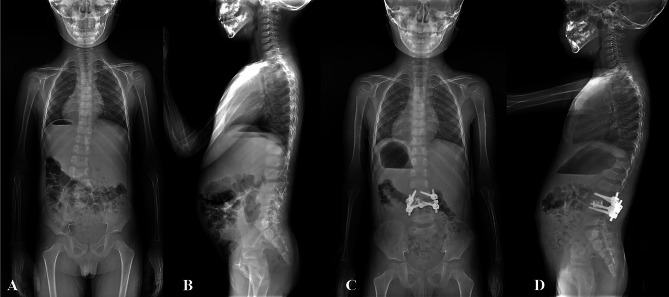
A case of partially segmented hemivertebra with concomitant cardiac abnormalities A 5/M, diagnosed with congenital scoliosis, he had a partially segmented hemivertebra at L3 segment **(a, b).** The ultrasonic cardiography of him showed mitral valve redundancy. And he underwent posterior hemivertebra resection and short segment fusion surgery, the X-ray showed L3 hemivertebra was totally resected **(c, d)**

**Fig. 3:**
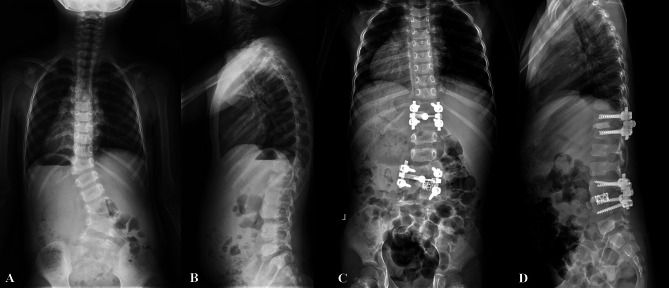
A case of mixed segmented hemivertebra with concomitant cardiac abnormalities A 4/F, diagnosed with congenital scoliosis, she had a fully segmented hemivertebra at T12 segment and partially segmented hemivertebra at L3/4 segment **(a, b).** Her ultrasonic cardiography result showed mitral valve redundancy and prolapse. And she underwent a 2-staged surgery, the X-ray showed hemivertebra at T12 and L3/4 was totally resected **(c, d)**

A total of 329 patients were enrolled, including 216 CS patients with hemivertebra and 113 patients without hemivertebra. In hemivertebra group, the mean age was 8.1 ± 5.0 years (range, 1.6–19 years) and the mean coronal Cobb angle of the major curve was 54.8 ± 12.2 degrees (range, 30 to 110 degrees). According to McMaster’s classification of hemivertebra, 152 patients had single hemivertebra, of which 94 cases were fully segmented (Fig. 1) and 58 cases were partially segmented (Fig. 2). The other 64 cases were multiple hemivertebra. Among the 64 cases with multiple hemivertebra, 24 cases were all fully segmented, 21 cases were all partially segmented, and the other 19 cases were both fully segmented and partially segmented(mixed) (Fig. 3). In non-hemivertebra group, including 40 males and 73 females, with an average age of 12.5 ± 3.5 years (range, 3–20 years). The mean coronal Cobb angle of their main curve was 61.3 ± 17.3 degrees (range, 26 to 108 degrees).

### UCG observation items

The results of UCG were recorded. The abnormalities were divided into two main types: valvular lesions and non-valvular lesions. Valvular lesions include mitral valves, tricuspid valves, aortic valves and pulmonary valves with redundancy, prolapse, insufficiency, stenosis and other abnormalities. Non-valvular lesions mainly refer to abnormalities other than the above-mentioned valve lesions, which include atrial septal defects, patent foramen ovale, ventricular septal defects, patent ductus arteriosus, and atrial or ventricular diameter abnormalities (except valve insufficiency or stenosis).

### Statistical analysis

SPSS 17.0 for Windows was used for statistical analysis. Student’s T test was used for the comparison of continuous variables, and the chi-square test and ANOVA test was used for categorical variables. A difference was statistically significant if P < 0.05.

## Results

### Comparison of the UCG findings between CS patients with hemivertebra or without hemivertebra

A total of 329 patients were analyzed, including 216 patients with hemivertebra and 113 patients without hemivertebra. In hemivertebra group, 61 (28.2%) cases had abnormal UCG results, and 31 (14.3%) were male and 30 (13.9%) were female. In non-hemivertebra group, 28(24.8%) cases had abnormal UCG results. No significant difference in the incidence of UCG between hemivertebra group and non-hemivertebra group (P = 0.517). There were no significant differences in sex, curve direction or location between groups (P > 0.05) (Table 1).

**Table 1:** Comparison of the occurrence of normal and abnormal UCG founding between CS patients with hemivertebra or without hemivertebra

		HV group		Non-HV group		P value
		Abnormal UCG	Normal UCG	Abnormal UCG	Normal UCG	
Total		61	155	28	85	0.517
Gender	Male	31	92	10	30	1.000
	Female	30	63	18	55	0.306
Curve direction	Left side	32	77	9	34	0.319
	Right side	29	78	19	51	1.000
Curve location	Thoracic	34	78	24	62	0.754
	Lumbar	27	77	4	23	0.311

HV: hemivertebra; UCG: ultrasonic cardiography;

### Comparison of the UCG findings between patients with single hemivertebra and multiple hemivertebra

There was no significant difference in the incidence of UCG abnormalities between single and multiple hemivertebra groups (P = 0.246). Binary logistic regression analysis showed that female sex with multiple hemivertebra was a risk factor for abnormal UCG (P = 0.009, OR = 3.449). There were no significant differences in sex, curve direction or location between groups (P > 0.05) (Table 2).

**Table 2:** Comparison of the occurrence of normal and abnormal UCG founding between CS patients with single or multiple hemivertebra

		Single HV		Multiple HV		P value
		Abnormal UCG	Normal UCG	Abnormal UCG	Normal UCG	
Total		39	113	22	42	0.246
Gender	Male	22	60	9	32	0.662
	Female	17	53	13	10	0.009
Curve direction	Left Side	24	55	8	22	0.816
	Right side	15	58	14	20	0.085
Curve location	Thoracic	22	54	12	24	0.664
	Lumbar	17	59	10	18	0.209

HV: hemivertebra; UCG: ultrasonic cardiography;

### Comparison of the UCG findings between patients with fully, partially and mixed segmented hemivertebra

There was no statistically significant difference in the incidence of UCG abnormalities between patients among fully, partially and mixed segmented hemivertebra group(P = 0.264). No significant difference in sex, curve direction or curve location among the three groups (P > 0.05) (Table 3).

**Table 3:** Comparison of the occurrence of normal and abnormal UCG founding between CS patients with fully, partially and mixed segmented hemivertebra

		Fully segmented HV		Partially segmented HV		Mixed segmented HV		P value
		Abnormal UCG	Normal UCG	Abnormal UCG	Normal UCG	Abnormal UCG	Normal UCG	
Total		30	88	26	53	5	14	0.264
Gender	Male	13	57	14	25	4	10	0.134
	Female	17	31	12	28	1	4	0.710
Curve direction	Left side	18	45	13	22	1	10	0.157
	Right side	12	43	13	31	4	4	0.243
Curve location	Thoracic	14	40	17	32	3	6	0.613
	Lumbar	16	48	9	21	2	8	0.789

HV: hemivertebra; UCG: ultrasonic cardiography;

### The details of valvular and non-valvular lesions in CS patients with hemivertebra

Among the 61 hemivertebra patients with abnormal UCG results, 33 of them with non-valvular lesions. Septal defects, which included atrial septal defects/patent foramen ovale and ventricular septal defects, composed nearly half of the abnormalities (48.5%, 16/33) (Table 4). 28 had valvular lesions. The main valvular abnormalities were mitral valve redundancy, mitral valve prolapse and mitral valve insufficiency (82.1%, 23/28) (Table 5). In all 216 hemivertebra patients, no significant difference existed between the rate of valvular non-valvular lesions (P = 0.581), or in sex, curve direction, curve location or segmentation of the HV between valvular lesion and non-valvular lesion group(P > 0.05) (Table 6).

**Table 4:** The details of hemivertebra patients with non-valvular lesions

		ASD	PFO	VSD	ASD + VSD	Chamber enlargement	coronary sinus dilatation	Other	Total
Total		2/2	4/2	2/2	1/1	5/4	4/1	1/2	19/14
Fully segmented HV	M/F	1/1	0/0	2/2	1/0	4/2	2/1	0/2	10/8
Partially segmented HV	M/F	0/1	2/2	0/0	0/1	1/1	2/0	1/0	6/5
Mixed segmented HV	M/F	1/0	2/0	0/0	0/0	0/1	0/0	0/0	3/1

HV: hemivertebra; M: male; F: female; ASD: atrial septal defect; PFO: patent foramen ovale; VSD: ventricular septal defect

**Table 5:** The details of hemivertebra patients with valvular lesions

		Mitral valve redundancy	Mitral valve redundancy and insufficiency	Mitral valve redundancy and prolapse	Mitral valve redundancy, prolapse and insufficiency	Mitral valve insufficiency	Aortic valve insufficiency	Bicuspid aortic valve and insufficiency	Mitral valve and tricuspid valve abnormality	Total
Total		9	2	5	1	6	2	1	2	28
Fully segmented HV	M/F	0/3	0/1	0/1	0/1	3/0	0/1	0/1	0/1	3/9
Partially segmented HV	M/F	5/1	0/1	1/2	0/0	1/2	1/0	0/0	0/1	8/7
Mixed segmented HV	M	0/0	0/0	1/0	0/0	0/0	0/0	0/0	0/0	1/0

HV: hemivertebra; M: male; F: female;

**Table 6:** Comparison of valvular and non-valvular lesions in CS patients with hemivertebra

	valvular lesions	non-valvular lesions	P value
Total	28	33	0.581
Male(123)	12	19	0.249
Female(93)	16	14	0.842
Left side curve(109)	15	17	0.851
Right side curve(107)	13	16	0.690
Thoracic curve(112)	15	19	0.577
Lumbar curve(104)	13	14	1.000
Fully segmented(118)	12	18	0.329
Partially segmented(79)	15	11	0.520
Mixed segmented(19)	1	4	0.340

## Discussion

The exact cause of hemivertebra deformity is not clear, but abnormal embryonic development can be one factor. During embryonic development, the skeletal system, cardiac and genitourinary system are of the same origin, so hemivertebra deformity patients may accompanied with cardiac abnormalities and urogenital abnormalities. Due to the development of only one side of the vertebral body, asymmetric growth forces may lead to scoliosis. Therefore, hemivertebra is the most common cause of congenital scoliosis [12]. Some studies have analyzed cardiac abnormalities using ultrasonic cardiography in patients with congenital scoliosis (CS) [11, 13−15]. However, there is still a lack of studies in patients with hemivertebra.

Zelop, Weisz, Wax and colleagues reported that fetal hemivertebra malformations found in prenatal ultrasound examinations are often combined with cardiac abnormalities [10, 16−17]. Goldstein et al. reported the prenatal diagnosis of 3 cases of hemivertebra malformation, and they found that among 78,500 infants born in Rambam Medical Center, Haifa, over 17 years (1985–2001), 26 were diagnosed with hemivertebra (for an incidence rate of 0.33/1000), and 8 of those cases were complicated with cardiac abnormalities (30.8%) [1]. In 2010, Bollini et al. analyzed 75 consecutive cases of hemivertebra, among which 6 cases (8%) were accompanied by cardiac abnormalities [13]. According to Basude et al., 4 (8.5%) of 47 children with hemivertebra had cardiac abnormalities [4]. In 2019, Passias et al. analyzed the data of 12,039,432 hospitalized children in the American Kids Inpatient Database (KID). They reported that the weighted incidence rate of hemivertebra deformity was 9.1 (8.6–9.6) per 100,000 patients. The patients with hemivertebra had the highest incidence of concurrent non-spine system anomalies, and the incidence of cardiac anomalies was 37.1% (34.7-39.5%). In their study, the incidence of cardiac abnormalities in patients with hemivertebra was more than twice as high as previously reported [5]. In the current study, the incidence of abnormal UCG results in children with hemivertebra was 28.2% (61/216), which was similar to that reported by Goldstein and Passias et al. [1, 5] but higher than that reported by Bollini and Basude et al. [3, 4]. There was no significant difference in the incidence of UCG between the patients with congenital scoliosis with and without hemivertebra (P = 0.517), unlike what was reported by Passias et al. [5].

Because of the low incidence rate of hemivertebra malformation and even lower incidence of concurrent cardiac anomalies, few related factors have been found in the literature. The current study showed that sex, number of hemivertebra (single vs. multiple), segmentation of the hemivertebra, curve direction and location did not affect the occurrence of cardiac abnormalities. This is consistent with the report by Bollini et al. [3]. However, according to Bollini et al., the incidence of cardiac abnormalities in patients with thoracic hemivertebra was significantly higher than that in patients with lumbar vertebrae (P = 0.05), which was not found in the current study. The current study also showed that female sex with multiple hemivertebra was a risk factor for UCG abnormalities, which was not reported in the earlier literature.

Among the types of cardiac abnormalities, non-valvular lesions are the main ones reported [1–5]. In 2006, Forrester et al. analyzed 316,508 infants born in Hawaii from 1986 to 2002 and found that 42 had hemivertebra malformations. Among them, ventricular septal defects (9 cases), atrial septal defects (5 cases), persistent left superior vena cava (3 cases), tetralogy of Fallot (2 cases) and anomalous pulmonary venous return (2 cases) were the most common cardiac abnormalities [2]. Passias et al. analyzed the clinical data of patients with vertebral abnormalities such as block vertebra, hemivertebra and vertebral body loss. The results showed that 30.3% of patients had cardiac abnormalities. The most common congenital heart malformations were atrial septal defects (12.3%), patent ductus arteriosus (10.4%), ventricular septal defects (8.7%), tetralogy of Fallot (2.6%) and aortic valve insufficiency (1.7%) [18]. There were 33 cases of non-valvular lesions in the current study, and the most common ones were atrial septal defects, patent foramen ovale and ventricular septal defects (48.5%, 16/33).

There are few reports about valve lesions on the UCG of patients with hemivertebra malformations. Forrester et al. reported that 1 of 42 patients with hemivertebra malformation had pulmonary atresia and stenosis (2.38%, 1/42) [2]. In the report of Passias et al., the weighted incidence of congenital aortic insufficiency was 1.68% [18]. In the current study, 28 patients had valvular abnormalities, of which mitral valve redundancy, mitral valve prolapse and mitral valve insufficiency (82.1%, 23/28) were the main ones. There was no significant difference between the incidence of non-valvular and valvular abnormalities, which was different from the lower incidence of valve abnormalities reported in the literature. Yazawa reported that the development of three-dimensional spinal deformity can lead to thoracic deformation, which will affect the structure and function of the heart and cause cardiac valve abnormalities [19]. The different results of the current study may be related to the older age of our patients.

This study had some limitations. First, its retrospective nature gives it inherent shortcomings. Second, in the patient group with valvular abnormalities, we included patients with simple mitral valve redundancy (9 patients), which may not have been analyzed in some studies. Third, this study only analyzed surgically treated patients with hemivertebra, and those without surgical treatment were not included.

## Conclusion

In summary, to our knowledge, this is the largest case study of UCG analysis in patients with congenital scoliosis caused by hemivertebra deformity. In 216 patients with hemivertebra, the incidence of abnormal UCG results was 28.2%. The number of hemivertebra, the segmentation and the curve direction and location did not affect the occurrence of UCG abnormalities. Female sex along with multiple hemivertebra was a risk factor for UCG abnormalities. In patients with UCG abnormalities, mitral valve abnormalities are the most common type.

## Data Availability

The datasets used and/or analysed during the current. study are available from the corresponding author on reasonable request.

## Abbreviations

CS: Congenital scoliosis
UCG: ultrasonic cardiography
IRB: Institutional Review Board
KID: Kids Inpatient Database
